# Temporal trends in incidence and mortality of colorectal cancer in Denmark from 2007 to 2022

**DOI:** 10.1002/ijc.35400

**Published:** 2025-03-14

**Authors:** Ida Ravnsbæk Johannsen, Anders Kindberg Boysen, Frank V. Mortensen, Jakob Kirkegård

**Affiliations:** ^1^ Department of Surgery, HPB Section Aarhus University Hospital Aarhus Denmark; ^2^ Department of Clinical Medicine Aarhus University Aarhus Denmark; ^3^ Department of Oncology Aarhus University Hospital Aarhus Denmark

**Keywords:** cancer, colorectal cancer, epidemiology

## Abstract

Colorectal cancer (CRC) is the third most common cancer in the Western world and represents a significant burden on healthcare systems worldwide. We aimed to describe temporal trends in incidence, tumor characteristics, and survival for patients with CRC in a nationwide, population‐based cohort in Denmark. We used population‐based Danish healthcare registries to study all patients diagnosed with CRC from 2007 to 2022. Exactly 76,955 people in Denmark were diagnosed with CRC from 2007 to 2022. ASIRs were relatively stable from 2007 to 2013, with an ASIR of 65.8 per 100,000 for colon cancer and 32 per 100,000 for rectal cancer. In 2014, an increase in incidence was observed (79.8 per 100,000 for colon cancer and 37.4 per 100,000 for rectal cancer), followed by a decline in later years. Median survival times were 4.1 (IQR: 0.8 to 14.1) years for patients diagnosed between 2007 and 2010, 5.3 (IQR: 1.1 to —) years for patients diagnosed from 2011 to 2013, and 7.6 (IQR: 1.7 to —) years for patients diagnosed from 2014 to 2017. The assessment of mutational and molecular profiles increased consistently throughout the study period. We observed an initial increase in CRC incidence in 2014, corresponding with the implementation of the national screening program, followed by a subsequent decline. In recent years, the incidence has dropped below pre‐screening levels. Additionally, the increasing use of molecular and mutational profiling reflects the growing complexity and multidisciplinary nature of CRC management.

## Graphical abstract

What's new?

Colorectal cancer (CRC) is the third most common cancer in the Western world. Widespread use of screening programs has helped raise awareness of the disease and improve survival rates. To make sure screening, treatment, research, and education goals stay relevant, it is important to collect accurate population‐based estimates of CRC incidence, characteristics, and mortality. Here, the authors used data from Danish population‐based cancer registries to describe trends in CRC from 2007 to 2022. They report an overall decrease in incidence and an increase in survival, as well as an increasing use of molecular tools to profile tumors.

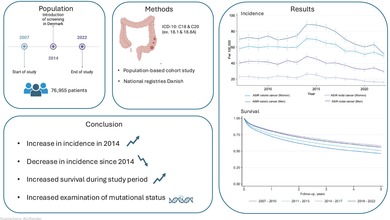

## 1 INTRODUCTION

Colorectal cancer (CRC) is the third most common cancer in the Western world and represents a significant burden on healthcare systems worldwide.^1^, ^2^ CRC is projected to affect 2.5 million new individuals worldwide by 2035, mainly due to lifestyle changes associated with economic progress in developing countries, an aging population, and dietary habits.^3^, ^4^ Incidence and survival vary globally, with the highest incidence and survival rates in developed countries.^3^

The development of CRC is multifactorial and depends on genetic, environmental, and dietary factors such as consumption of red meat, obesity, smoking, and alcohol.^5^, ^6^ Survival of CRC patients has improved, which is likely due to improved surgical techniques, more effective chemotherapy in both adjuvant and palliative settings, and increasing use of personalized treatments such as immunotherapy and targeted therapies.^7^, ^8^ Furthermore, the introduction of nationwide screening programs in an increasing number of countries worldwide has considerably enhanced disease awareness and improved survival rates.^9^

Accurate population‐based estimates of CRC incidence, characteristics, and mortality are essential for informing patients, healthcare professionals, and decision‐makers, and to identify areas for further research. The aim of this study was to describe temporal trends in incidence, tumor characteristics, and survival for patients with CRC in a nationwide, population‐based cohort in the Danish tax‐financed, universal healthcare system.

## 2 METHODS

### 2.1 Setting, study design, and data sources

We conducted a nationwide, population‐based cohort study of all patients registered with a diagnosis of CRC in either the Danish Cancer Registry (DCR) or the Danish National Patient Registry (DNPR) between 1 January 2007 and 31 December 2022. We obtained information from several nationwide registries, which can all be linked at an individual level using the Civil Personal Registration (CPR) number, which is assigned to all residents in Denmark at birth or immigration.^10^

The DCR was established in 1943 and contains information on the date of diagnosis, cancer site, histology, dissemination, and other variables for all cancers diagnosed in Denmark.^11^ The validity and completeness are high due to mandatory reporting and manual quality control routines in the daily production of the DCR.^11^

The DNPR contains information on all inpatient hospitalizations to Danish public hospitals since 1977, whilst outpatient and emergency room visits have been included since 1995.^12^ Since 1994, the International Classification of Diseases, 10th revision (ICD‐10) has been used. The validity for diagnoses in DNPR has been examined and generally found to have a positive predictive value above 80 and a good completeness.^12^

The Civil Registration System (CRS) contains administrative data on variables like birth date, sex, sequential dates of migration, and vital status for every resident in Denmark since 1968.^10^ It is updated daily and is virtually complete.

The Danish Colorectal Cancer Group Database (DCCD) was established in 1994 and contains information on all patients with rectal cancer diagnosed and/or treated at a surgical department. Since 2001, the database has also included information on all patients with colon cancer. It has details on surgical and oncological treatments, as well as variables providing information on pathology, patient characteristics, and risk factors such as smoking, alcohol consumption, and body mass index (BMI).^13^ Data from the DCCD was available through 2020. The database has >95% completeness and a high validity of data since most of the information is recorded by surgeons or pathologists.^13^

The Danish National Pathology Registry (DPR) holds information on all tissue examinations and pathology specimens examined at Danish hospitals since 1997. For CRC patients, the registry includes information on resection margins, histology, and mutational status.^14^ Mutational status has been registered systematically in CRC patients in Denmark since 2013.^15^, ^16^ Information in the DPR is a central daily routine diagnostic tool for pathologists. It is updated daily, and the level of missing data is expected to be extremely low.^14^

### 2.2 Study population

We identified all patients, aged 18+ years, diagnosed with CRC (ICD‐10: C18 and C20 excluding appendiceal cancer; C18.1 and hereditary non‐polyposis colorectal cancer; C18.8A) from 2007 to 2022. Patients were grouped into four different time periods depending on the year of diagnosis: 2007–2010, 2011–2013, 2014–2017, and 2018–2022. In case of different dates of diagnosis between the databases (DCR, DNPR), we used the date recorded in the DCR, as reporting of all malignancies to this registry is mandatory.

### 2.3 Information on CRC characteristics

Information on tumor stage was retrieved from the DCR, the DNPR, and the DCCD. The tumor stage was registered according to the tumor‐node‐metastasis (TNM) classification. TNM stage was recorded according to the existing edition at the time of diagnosis (6th edition until 2017 where the 8th edition was introduced^17^, ^18^). Tumor stage (Union for International Cancer Control, UICC^19^) was derived from the TNM classification. From the DCR and the DPR, we obtained information on tumor histology (adenocarcinoma, mucinous adenocarcinoma, signet ring cell carcinoma, and others). Details on the molecular biomarkers included the mutational status of the oncogenes BRAF and K/NRAS, and the level of microsatellite instability defined as either high level (MSI‐H) or low level (MSI‐L) were obtained from the DPR.

### 2.4 Information on comorbidities

From the DNPR, we obtained a full list of all registered comorbidities in individuals in the study population. This information was used to calculate the Charlson Comorbidity Index (CCI) score.^20^ We defined three levels of comorbidity: Low (score 0), moderate (score 1–2) and severe (score >2) comorbidity. We also computed the Nordic Multimorbidity Index (NMI) for the overall comorbidity burden for each patient. The NMI is a validated comorbidity index designed to predict 5‐year mortality in a Danish population.^21^ From the DCCD, information on risk factors such as smoking, alcohol, and BMI was obtained.

### 2.5 Information on cancer treatment

We ascertained information on cancer‐directed treatment from the DNPR. Surgery codes have been registered since 1977 and recorded according to the *Nordic Medico‐Statistical Committee Classification of Surgical Procedures*.^22^ Codes for oncological treatment have been registered since 2004. We captured all treatments, both oncological and surgical, registered within 30 days before and up to 180 days after CRC diagnosis. Treatment was classified as surgery, oncological treatment, targeted treatments with antibodies against the epidermal growth factor receptor (EGFR) or vascular endothelial growth factor receptor (VEGF), and best supportive care (defined as no records of cancer‐directed treatment within the capture period).

### 2.6 Follow‐up

Patients were followed from the date of CRC diagnosis to death, emigration, or August 18, 2024, whichever occurred first. The dates of death or emigration were obtained from the CRS.

### 2.7 Statistical analyses

We present descriptive characteristics as means with standard deviations or medians with interquartile ranges, where appropriate, and counts with percentages. For each year, we calculated the CRC incidence as the number of new diagnoses. They are presented as new cases per 100,000 population with age‐standardized incidence rates (ASIR), standardized to the European standard population (in 2010). Survival analyses were performed using the Kaplan–Meier estimator. We computed 1‐, 3‐, and 5‐year and median survival times. All estimates are presented with associated 95% confidence intervals. Statistical analyses were performed using STATA 18 (StataCorp, College Station, TX) The Joinpoint regression program (*Version 5.3.0.0*—*November 2024*), developed by the US National Cancer Institute (*Statistical Methodology and Applications Branch*, *Surveillance Research Program*, *National Cancer Institute*), was used to examine trends in incidence and to calculate the Annual Percent Change (APC).^23^, ^24^

### 2.8 Patient and public involvement (PPI)

We did not directly include PPI in this study, but one of the databases (DCCD) used in the study was developed with PPI and is updated by a committee that includes patient representatives.

## 3 RESULTS

### 3.1 Descriptive characteristics

We identified 76,955 patients diagnosed with CRC. Median age was 71 years from 2007 to 2017 and 73 years from 2018 to 2022. From 2007 to 2013, 52.6% of patients were male, increasing to 54.5% and 53.4% from 2014 to 2017 and 2018 to 2022, respectively. Comorbidity levels were stable in the different time periods of the study (Table 1).

**TABLE 1:** Descriptive characteristics of 76,955 Danish patients diagnosed with colorectal cancer during 2007–2022 (grouped by time of diagnosis).

	2007–2010	2011–2013	2014–2017	2018–2022
Total	17,550	13,889	22,433	23,083
Age, median (IQR)	71 (63–79)	71 (64–79)	71 (64–78)	73 (64–80)
Age group				
<50 years	799 (4.6%)	599 (4.3%)	851 (3.8%)	982 (4.3%)
50–59 years	2059 (11.7%)	1564 (11.3%)	2597 (11.6%)	2575 (11.2%)
60–69 years	4848 (27.6%)	3861 (27.8%)	6242 (27.8%)	5309 (23.0%)
70–79 years	5585 (31.8%)	4523 (32.6%)	7970 (35.5%)	8069 (35.0%)
+80 years	4259 (24.3%)	3342 (24.1%)	4773 (21.3%)	6148 (26.6%)
Sex				
Men	9244 (52.7%)	7307 (52.6%)	12,232 (54.5%)	12,325 (53.4%)
Women	8306 (47.3%)	6582 (47.4%)	10,201 (45.5%)	10,758 (46.6%)
Charlson Comorbidity Index				
Low (0)	11,823 (67.4%)	9163 (66.0%)	15,330 (68.3%)	15,849 (68.7%)
Moderate (1, 2)	4573 (26.1%)	3805 (27.4%)	5775 (25.7%)	5895 (25.5%)
Severe (>2)	1154 (6.6%)	921 (6.6%)	1328 (5.9%)	1339 (5.8%)
Nordic Multimorbidity Index, mean (SD)	7.9 (11.1)	8.0 (11.2)	7.0 (10.7)	7.5 (10.6)
Tobacco smoking				
Non‐smoker	3657 (20.8%)	3757 (27.1%)	7257 (32.3%)	4846 (21.0%)
Current smoker	2137 (12.2%)	2149 (15.5%)	3457 (15.4%)	1939 (8.4%)
Former smoker	4257 (24.3%)	4213 (30.3%)	6984 (31.1%)	4279 (18.5%)
Unknown	7499 (42.7%)	3770 (27.1%)	4735 (21.1%)	12,019 (52.1%)
Alcohol				
0	3065 (17.5%)	2891 (20.8%)	4198 (18.7%)	2941 (12.7%)
1–14	5338 (30.4%)	6014 (43.3%)	11,507 (51.3%)	6803 (29.5%)
>14	1580 (9.0%)	1339 (9.6%)	2220 (9.9%)	1317 (5.7%)
Unknown	7567 (43.1%)	3645 (26.2%)	4508 (20.1%)	12,022 (52.1%)
BMI mean (SD)	25.4 (4.3)	25.5 (4.3)	25.9 (4.5)	26.1 (4.7)
Missing	7746 (44.1%)	3424 (24.7%)	3776 (16.8%)	11,258 (48.8%)
cT‐stage				
cT1	939 (5.4%)	673 (4.8%)	2719 (12.1%)	2910 (12.6%)
cT2	1491 (8.5%)	1298 (9.3%)	2970 (13.2%)	3617 (15.7%)
cT3	7509 (42.8%)	5527 (39.8%)	8620 (38.4%)	9525 (41.3%)
cT4	3727 (21.2%)	2596 (18.7%)	3708 (16.5%)	4752 (20.6%)
cTx	3884 (22.1%)	3795 (27.3%)	4416 (19.7%)	2279 (9.9%)
cN‐stage				
cN0	6053 (34.5%)	4698 (33.8%)	9396 (41.9%)	10,360 (44.9%)
cN1	3351 (19.1%)	2353 (16.9%)	4139 (18.5%)	5845 (25.3%)
cN2	3060 (17.4%)	2111 (15.2%)	3290 (14.7%)	4184 (18.1%)
cN3	227 (1.3%)	100 (0.7%)	100 (0.4%)	37 (0.2%)
cNx	4859 (27.7%)	4627 (33.3%)	5508 (24.6%)	2657 (11.5%)
cM‐stage				
cM0	11,635 (66.3%)	9391 (67.6%)	16,422 (73.2%)	17,337 (75.1%)
cM1	4605 (26.2%)	3440 (24.8%)	4572 (20.4%)	4776 (20.7%)
cMx	1310 (7.5%)	1058 (7.6%)	1439 (6.4%)	970 (4.2%)
UICC stage				
Stage I	1982 (11.3%)	1756 (12.6%)	4760 (21.2%)	5176 (22.4%)
Stage II	4654 (26.5%)	3761 (27.1%)	5280 (23.5%)	4623 (20.0%)
Stage III	4573 (26.1%)	3372 (24.3%)	5525 (24.6%)	6938 (30.1%)
Stage IV	4843 (27.6%)	3582 (25.8%)	4720 (21.0%)	4792 (20.8%)
Unknown	1498 (8.5%)	1418 (10.2%)	2148 (9.6%)	1554 (6.7%)
Histology				
Adenocarcinoma	14,612 (83.3%)	11,513 (82.9%)	18,917 (84.3%)	19,476 (84.4%)
Muc. adenocarcinoma	1284 (7.3%)	1063 (7.7%)	1595 (7.1%)	1773 (7.7%)
Signet ring cell	206 (1.2%)	126 (0.9%)	182 (0.8%)	212 (0.9%)
Other	274 (1.6%)	247 (1.8%)	350 (1.6%)	449 (1.9%)
Unknown	1174 (6.7%)	940 (6.8%)	1389 (6.2%)	1173 (5.1%)
Tumor location				
Right‐sided colon	4215 (24.0%)	3542 (25.5%)	5693 (25.4%)	7297 (31.6%)
Transverse colon	973 (5.5%)	731 (5.3%)	1271 (5.7%)	1638 (7.1%)
Left‐sided colon	5167 (29.4%)	4087 (29.4%)	7228 (32.2%)	6026 (26.1%)
Rectum	5822 (33.2%)	4560 (32.8%)	6919 (30.8%)	7138 (30.9%)
Other/unknown	1373 (7.8%)	969 (7.0%)	1322 (5.9%)	984 (4.3%)
Serum CEA (μg/L), median (IQR)	5 (2–11)	5 (2–10)	3 (2–8)	3 (2–9)
Missing	16,558 (94.3%)	11,980 (86.3%)	16,062 (71.6%)	14,494 (62.8%)
Screening	—	—	4600 (20.5%)	4683 (20.3%)

*Note*: Data from the DCCD was available through 2020.

Abbreviations: IQR, interquartile range; SD, standard deviation.

### 3.2 Tumor characteristics

Among patients with a reported tumor location, left‐sided and rectal cancers were the most common (26.1% and 30.9% from 2018 to 2022, respectively). The proportion of histologically verified tumors was higher than 90% throughout the study period. Adenocarcinomas accounted for ~80% of the verified cases, and tumor staging was conducted in more than 90% of the patients. The distribution of tumor stage shifted toward a lower tumor stage during the study period, with 11.3% of patients being diagnosed with UICC stage I disease from 2007 to 2010 and 22.4% from 2018 to 2022, whereas patients diagnosed with UICC stage IV disease decreased, from 27.6% to 20.8% (Table 1). The frequency of patients with unknown T‐ and N‐stage declined during the study period.

### 3.3 Incidence

**FIGURE 1:**
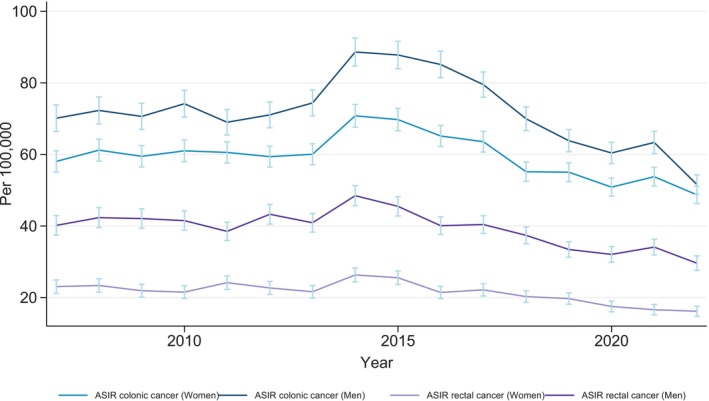
Age‐standardized incidence rates per 100,000 population (standardized to the European standard population from 2010) of colon and rectal cancer for men and women in Denmark from 2007 to 2022. *Range of error bars: 95% CI.

**FIGURE 2:**
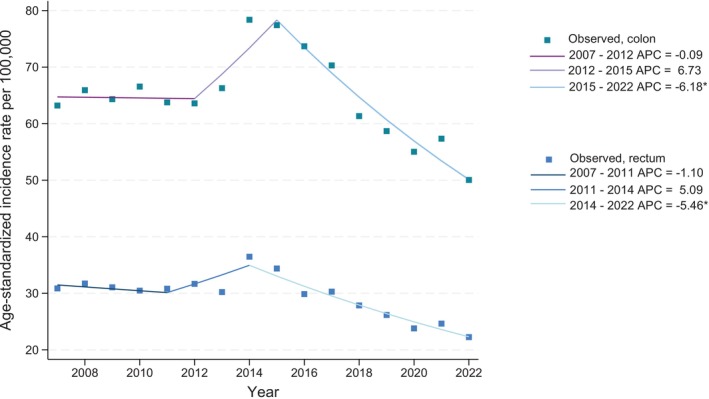
Joinpoint regression model for colon and rectal cancer incidence rates. *Indicates that the Annual Percent Change (APC) is significantly different from zero at the alpha = 0.05 level.

The incidence of CRC in Denmark remained relatively stable from 2007 to 2013, with an ASIR of 65.8 per 100,000 for colon cancer and 32 per 100,000 for rectal cancer. An increase in incidence was observed in 2014 (79.8 per 100,000 for colon cancer and 37.4 per 100,000 for rectum), after which the incidence decreased (Figure 1). We found a higher incidence in males compared with females for both colon and rectal cancer throughout the study period (Figure 1). Using the Joinpoint regression model, we observed a stable Annual Percent Change (APC) for both colon and rectal cancer at the beginning of the study period. For colon cancer, the APC was 6.73% (95% CI: −10.09% to 10.48%) from 2012 to 2015 and −6.18% (95% CI: −9.63% to −0.76%) from 2015 to 2022. For rectal cancer, the APC was 1.23% (95% CI: −8.30% to 8.20%) from 2011 to 2014 and −5.46% (95% CI: −9.95% to −1.30%) from 2014 to 2022 (Figure 2).

### 3.4 Mutational status

From 2013 to 2022, a total of 50,191 patients were diagnosed with CRC. Among these, 90.1% of patients had information available for the molecular biomarkers. Exactly 7.8% had a BRAF mutation, 20.8% had a K/NRAS mutation, and 17.5% had MSI‐H status. Most patients with a K/NRAS mutation were male (57.1%), while most patients with a BRAF mutation and MSI‐H were female (62.5% and 62.5%, respectively).

### 3.5 Treatment

**FIGURE 3:**
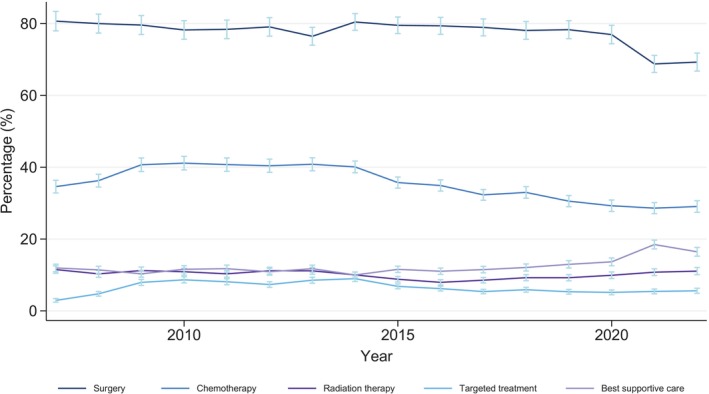
Percentage with 95% CI of patients with CRC treated with: Surgery, Chemotherapy, Radiation therapy, Targeted treatment, and Best supportive care in Denmark from 2007 to 2022. *Range of error bars: 95% CI.

During the study period, 59,824 (77.7%) patients received surgical treatment for their CRC (Figure 3). Of these, 37,067 (48.2%) did not receive supplementary oncological treatment. Chemotherapy alone was administered to 2554 (3.3%) of the patients. Targeted treatments were administered to 4988 (6.5%) patients, and 1399 (1.8%) patients were treated with radiotherapy alone. A group of patients was not deemed fit for any active treatment and received the best supportive care (9438 [12.3%]). From 2014, a small decline was observed in all treatment modalities within the first 180 days after diagnosis (Figure 3).

### 3.6 Survival

#### 3.6.1 Entire population

**FIGURE 4:**
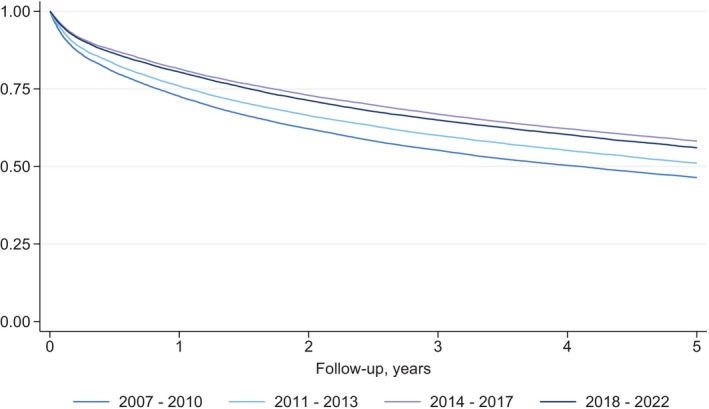
Kaplan–Meier survival estimates of patients with CRC in Denmark diagnosed from 2007 to 2022.

For the entire population, the survival increased during the study period (Figure 4). The 1‐year survival was 72.6% (95% CI: 71.9%–73.3%) from 2007 to 2010 compared with 80.4% (95% CI: 79.9%–80.9%) from 2018 to 2022. Similarly, the 5‐year survival increased from 46.4% (95% CI: 45.7%–47.1%) from 2007 to 2010 to 56.0% (95% CI: 55.3%–56.7%) from 2018 to 2022. Median survival time was 4.1 (IQR: 0.8–14.1) years for patients diagnosed from 2007 to 2010, 5.3 (IQR: 1.1 to —) years for patients diagnosed from 2011 to 2013, and 7.6 (IQR: 1.7 to —) years for patients diagnosed from 2014 to 2017.

#### 3.6.2 According to mutational statuses

**FIGURE 5:**
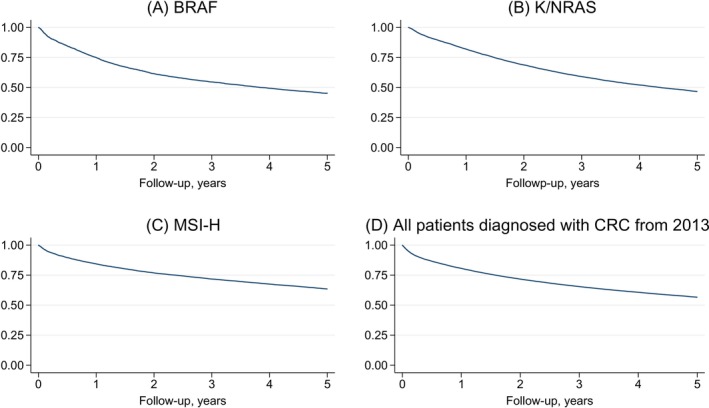
Kaplan–Meier survival estimates for patients with CRC diagnosed from 2013 to 2022. (A) Kaplan–Meier estimates for patients with BRAF mutation, (B) Kaplan–Meier estimates for patients with K/NRAS mutation, (C) Kaplan–Meier estimates for patients with MSI‐H. (D) Kaplan–Meier estimates for all patients diagnosed with CRC from 2013 to 2022.

We found a 1‐year survival of 80.6% (95% CI: 80.2%–81.0%) for all patients diagnosed since 2013, irrespective of mutational status. For patients with at least one known mutation, the 1‐year survival was 74.9% (95% CI: 73.5% –76.2%) for BRAF mutations, 81.9% (95% CI: 81.2%–82.7%) for a K/NRAS mutation, and 77.2% (95% CI: 71.3%–82.1%) for patients with both BRAF and K/NRAS mutations (Figure 5). The 5‐year survival was 56.6% (95% CI: 56.1%–57.0%) for the entire population, 45.2% (95% CI: 43.5%–46.8%) for patients with a BRAF mutation, and 46.6% (95% CI: 45.6%–47.6%) for K/NRAS mutations. For patients with MSI‐H, the survival rates were higher than for the entire population, with a 1‐ and 5‐year survival of 84.3% (95% CI: 83.5%–85.1%) and 63.5% (95% CI: 62.4%–64.5%), respectively (Figure 5).

## 4 DISCUSSION

In the present study of all patients diagnosed with CRC in Denmark from 2007 to 2022, we report a decreasing incidence and improved survival. We also report an increasing examination of the molecular status in patients with CRC.

We observed age‐standardized incidence rates of CRC in Denmark of 57.3 per 100,000 for colon cancer and 25.7 per 100,000 for rectal cancer in the years from 2018 to 2022. To our knowledge, no studies to date have reported on such recent data. The incidence rates from 2007 to 2017 are in agreement with other studies on European^25^ and US^26^ populations. We observed a notable increase in incidence around 2014, which is widely attributed to the introduction of the fecal immunochemical test (FIT)‐based screening program for CRC in Denmark. Numerous studies have demonstrated that screening programs reduce CRC incidence and mortality, especially in countries that have introduced a nationwide program as promptly as in Denmark.^27^, ^28^, ^29^, ^30^, ^31^ This is further supported by both the decreasing incidence observed in the years following the introduction of the screening program (from 2015 to 2018) and a relatively stable incidence rate hereafter (from 2019 to 2021). Year 2020 was the most impactful year for the COVID‐19 pandemic, during which a decrease in incidence was expected. According to the Danish Health Data Authority, the incidence rate of colon cancer decreased by 8.0% for women and 3.9% for men compared with 2019. For rectal cancer, the decrease was 7.4% for men and 8.0% for women.^32^ The subsequent increase in 2021, surpassing 2019 levels, could be attributed to the pandemic, with more patients postponing visits to their doctor. Overall, cancer incidence in Denmark dropped by 0.6% in 2020 compared with the previous year.^32^

The decrease in incidence since 2014 is likely attributable to the increase in colonoscopies following the introduction of the program, which has led to more polypectomies with the removal of pre‐cancerous lesions.^33^ Recently, the participation in the national screening program has been decreasing, which has led to concerns about a likely increase in incidence in the coming years.^34^

Curative‐intent surgery is the primary treatment modality for localized CRC (UICC stages I and II). Patients with small localized rectal cancers (UICC stages I and II) can, in some cases, undergo non‐operative treatment with definitive chemoradiotherapy.^35^ The proportion of patients undergoing surgery was stable throughout the study but decreased slightly in 2021 and 2022, where we also observed a concomitant increase in patients receiving only best supportive care. This may be caused by the COVID‐19 pandemic, contributing to diagnostic delays, leading to patients being diagnosed in more advanced disease stages. We also saw a decrease in the use of chemotherapy within 180 days after CRC diagnosis. This may be explained by the increasing proportion of low‐stage tumors, as peri‐operative or adjuvant chemotherapy is only administered in locally advanced cancers.^36^, ^37^ Immunotherapy was administered to 159 patients during our study. This group of therapeutic agents received regulatory approval in Denmark in 2019 for patients with metastatic disease and MSI‐H genotype, hence only covering the final years of our cohort.

The 1‐ and 5‐year survival of CRC increased from 72.6% and 46.6%, respectively, from 2007 to 2010 to 80.4% and 56.0% from 2018 to 2022. An increase in survival is expected with the introduction of a new diagnostic tool as a national screening program.^38^ Furthermore, advancements in multidisciplinary patient care and new surgical and oncological therapies likely also contributed to improved survival. The highest survival rate was seen for patients diagnosed from 2014 to 2017, with a 1‐year survival of 81.5% and a 5‐year survival of 58.2%. The observed peak survival is likely attributable, at least in part, to lead time bias. This bias occurs because patients are being diagnosed with CRC earlier than before the introduction of the screening program, thereby contributing to an inflated survival time.

In recent decades, the incidence and mortality rates of CRC have generally declined in the Western world,^25^ more prominently in countries with established screening programs.^39^ In Denmark, CRC mortality has aligned with that of other Nordic countries that either have not introduced screening programs yet (Sweden) or have only recently implemented one (Norway in 2022).^25^, ^40^, ^41^ While one might expect the CRC mortality and incidence rates to be lower compared with other Nordic countries, the delay in seeing this effect may be attributed to Denmark's historically higher mortality rates before 2014.

We found information about mutational status available in 90.1% of patients diagnosed since 2013. This is consistent with the number of patients undergoing biopsy or surgical treatment. Information on K/NRAS and BRAF mutations was less complete than information on MSI status, as analysis of MSI status is regarded as essential for all newly diagnosed patients with CRC, while the analysis of K/NRAS and BRAF mutations is made upon request, serving to individualize the oncological treatment course. The distribution of mutations reported in our study correlates well with other studies.^42^, ^43^ Survival analysis based on molecular status gives insight into the prognostic value of underlying disease biology, as we report an increased mortality for BRAF and K/NRAS mutations compared with the general CRC population, in line with previous reports.^44^, ^45^ Furthermore, we report a survival benefit for the MSI‐H genotype, a subgroup of CRC patients known to have an improved prognosis and a superior treatment outcome for immunotherapy.^45^

The data collected in a uniform, tax‐financed healthcare system in combination with mandatory reporting of cancers and a virtually complete follow‐up contributes to a high validity of our findings. It should be noted, however, that there is some risk of underreporting due to limited workup in some patients, particularly the old and frail or very severely ill and metastatic patients.

## 5 CONCLUSION

We observed an increase in CRC incidence around the same time as the introduction of the national screening program and a decreasing incidence hereafter. In the most recent years, the incidence was lower than before the introduction of the screening program. We also report an improving survival. We found an increased examination of the mutational and molecular profiles of CRC, supporting the complex and multidisciplinary treatment landscape of CRC.

## AUTHOR CONTRIBUTIONS

**Ida Ravnsbæk Johannsen:** Conceptualization; investigation; writing – original draft; methodology; visualization; writing – review and editing; formal analysis. **Anders Kindberg Boysen:** Supervision; writing – review and editing; conceptualization. **Frank V. Mortensen:** Conceptualization; writing – review and editing; supervision. **Jakob Kirkegård:** Conceptualization; funding acquisition; writing – review and editing; visualization; methodology; supervision; data curation; formal analysis.

## FUNDING INFORMATION

Aage og Johanne Louis‐Hansens Fond, Tømmerhandler Vilhelm Bangs Fond, and Købmand Sven Hansen og Hustru Ina Hansens Fond. The study sponsors had no involvement in the design, conduct, analysis, or reporting of this study.

## CONFLICT OF INTEREST STATEMENT

All authors have completed the ICMJE uniform disclosure form at http://www.icmje.org/disclosure‐of‐interest/ and declare: no support from any commercial entity for the submitted work; no financial relationships with any commercial entities that might have an interest in the submitted work in the previous 3 years; and no other relationships or activities that could appear to have influenced the submitted work.

## ETHICS STATEMENT

This study was approved by the Danish Data Protection Agency (j.nr. 1‐16‐02‐105‐22). No ethical approval is required for register‐based studies in Denmark. All authors had access to the study data and reviewed and approved the final manuscript.

## Data Availability

The data can be accessed through an application at the Danish Health Data Authority (www.sundhedsdatastyrelsen.dk). Further information is available from the corresponding author upon request.
